# Distinct Roles for CXCR6^+^ and CXCR6^−^ CD4^+^ T Cells in the Pathogenesis of Chronic Colitis

**DOI:** 10.1371/journal.pone.0065488

**Published:** 2013-06-19

**Authors:** Yasushi Mandai, Daisuke Takahashi, Koji Hase, Yuuki Obata, Yukihiro Furusawa, Masashi Ebisawa, Tomoo Nakagawa, Toru Sato, Tatsuro Katsuno, Yasushi Saito, Takeshi Shimaoka, Osamu Yokosuka, Kotaro Yokote, Hiroshi Ohno

**Affiliations:** 1 Department of Clinical Cell Biology, Graduate School of Medicine, Chiba University, Chiba, Japan; 2 Department of Immune Regulation, Graduate School of Medicine, Chiba University, Chiba, Japan; 3 Department of Medicine and Clinical Oncology, Graduate School of Medicine, Chiba University, Chiba, Japan; 4 Laboratory for Epithelial Immunobiology, Research Center for Allergy and Immunology, RIKEN Yokohama Institute, Yokohama, Kanagawa, Japan; 5 Laboratory for Bioenvironmental Epigenetics, Research Center for Allergy and Immunology, RIKEN Yokohama Institute, Yokohama, Kanagawa, Japan; 6 Division of Immunobiology, Department of Supramolecular Biology, Graduate School of Nanobioscience, Yokohama City University, Yokohama, Kanagawa, Japan; 7 Division of Mucosal Barriology, International Research and Development Center for mucosal vaccines, The Institute of Medical Science, The University of Tokyo, Minato-ku, Tokyo, Japan; 8 Department of Molecular Preventive Medicine, Graduate School of Medicine, The University of Tokyo, Bunkyo-ku, Tokyo, Japan; Massachusetts General Hospital, United States of America

## Abstract

CD4^+^ T cells play a central role in the development of inflammatory bowel disease (IBD) via high-level production of effector cytokines such as IFN-γ and TNF-α. To better characterize the colitogenic CD4^+^ T cells, we examined their expression of CXCR6, a chemokine receptor that is expressed by T cells upon activation and is upregulated in several inflammatory diseases. We found that 80% of colonic lamina propria CD4^+^ T cells expressed CXCR6 in the CD45RB^high^ T cell-transferred colitis model. CXCR6 expression was similarly upregulated in inflamed mucosa of patients with Crohn’s disease. Although surface marker analysis demonstrated that both CXCR6^+^ and CXCR6^−^ CD4^+^ T-cell subsets consist of the cells with effector and effector-memory cells, the more cells in the CXCR6^+^ subset produced IFN-γ and TNF-α compared to CXCR6^−^ subset, and only the CXCR6^+^ subset produced IL-17A. Nevertheless, adoptive retransfer of lamina propria CXCR6^+^ T cells into *Rag1* ^−/−^ recipients failed to induce the disease due to limited expansion of the transferred cells. By contrast, retransfer of CXCR6^−^ cells evoked colitis similar to that observed in CD4^+^CD45RB^high^ T cell-transferred mice, and resulted in their conversion into CXCR6^+^ cells. Collectively, these observations suggest that the CXCR6^+^CD4^+^ T-cell subset consists of terminally differentiated effector cells that serve as the major source of effector cytokines in the inflamed tissue, whereas CXCR6^−^CD4^+^ T-cell subset serves as a colitogenic memory compartment that retains the ability to proliferate and differentiate into CXCR6^+^CD4^+^ T cells.

## Introduction

Inflammatory bowel disease (IBD), e.g., Crohn’s disease (CD) and ulcerative colitis (UC), are chronic and relapsing inflammatory disorders of the gastrointestinal tract. The chronic inflammation in the gut results from an excessive immune response to commensal microbiota [1]. Although multiple factors including genetic predisposition and environmental factors such as gut microbial composition have been implicated in the etiology of IBD, most of these factors converge on a common effector pathway, the generation of effector CD4^+^ T cells accumulating in the gut that orchestrate a persistent inflammatory response by producing inflammatory cytokines [1], [2]. The colitogenic CD4^+^ effector memory T cells are most likely generated during the onset of the inflammatory response, and continuously circulate throughout the body, as evidenced by the fact that CD4^+^ T cells isolated from peripheral blood of colitic mice can transfer the disease to healthy recipients [3], [4]. Although recirculating, these pathogenic effector memory CD4^+^ T cells reside preferentially in the bone marrow (BM) [5], [6], where IL-7 produced by stromal cells is considered a key cytokine involved in promoting their survival for long periods [7]. These colitogenic CD4^+^ T cells in the BM may eventually repopulate the intestine, where they give rise to effector CD4^+^ T cells after encountering intestinal antigens [7], [8]. Based on this model, colitogenic CD4^+^ effector-memory T cells may be divided into two distinct populations, i.e., cytokine-producing effector cells and long-term surviving memory cells that are quiescent but retain the ability to induce colitis upon activation. However, the identity of these colitogenic CD4^+^ effector and effector-memory T cells remains to be fully characterized.

Chemokines facilitate leukocyte migration and retention in lymphoid and peripheral non-lymphoid tissues. More than 40 chemokines have been identified so far, and they are classified into C, CC, CXC, and CX_3_C subfamilies based on the spacing of the first two amino terminal cysteine residues. The expression of several chemokines increases in the inflamed colon of patients with IBD [9], [10]. Recently, the expression of CXCL16, a CXC family chemokine, has been reported to be upregulated in the colon of CD patients and in mouse models of colitis [3], [5]. Furthermore, administration of an anti-CXCL16 mAb ameliorates inflammation in a chemically induced experimental colitis model. Thus, multiple lines of evidence suggest that the CXCL16-CXCR6 system may play an important role in colonic inflammation and could be a therapeutic target for CD [5], although the expression profile of CXCR6 on the colitogenic CD4^+^ T cells remains to be characterized.

We here report that CD4^+^ T cells in the inflamed colon of CD4^+^CD45RB^high^ T cell-transferred colitis model can be divided into two subpopulations according to the expression of CXCR6. The colonic CXCR6^+^ subset serves as a *bona fide* effector by preferentially producing IFN-γ, IL-17A and TNF-α. On the other hand, the CXCR6^−^ subset possesses a more limited ability to produce these cytokines but retains the capability to proliferate and convert to CXCR6^+^ cells after activation. Given that only the CXCR6^−^ subset can transfer the disease to recipient mice, this subset likely functions as the colitogenic CD4^+^ memory T cells that are responsible for the recurrence of inflammatory responses in IBD.

## Materials and Methods

### Colonic Biopsy Specimens

Biopsy specimens were obtained by endoscopy from inflamed areas of the colon of 6 patients with CD and 10 with UC, with the patients’ informed consent. Samples of normal controls (NC) were taken from 5 patients with colonic polyps and were free of inflammation histopathologically. The mean ± SEM (range) age of the patients with CD was 27.3 ± 3.7 (29 – 42) years, while that of UC was 36.6 ± 3.7 (24 – 61) years and that of NC was 55.8 ± 7.0 (33 – 73) years. Clinical activity was evaluated by serum concentration of C-reactive protein, CD Activity Index (CDAI) for patients with CD, and Lichtiger index (UCAI) for UC. Endoscopic activity was evaluated by Simple Endoscopic Score for CD (SES-CD), and Matts classification score for UC patients, respectively. The disease activity of the patients with CD was mild, as the mean ± SEM (range) of CRP was 1.57 ± 0.68 (0.4 – 4.8) mg/L, CDAI was 144.0 ± 45.6 (24.3 – 350.3), and SES-CD was 22.2 ± 6.2 (6 – 48). The activity of the UD patients ranged from remission to severe, as the mean ± SEM (range) of CRP was 2.12 ± 0.85 (0.1 – 6.7) mg/mL, UCAI was 7.9 ± 1.5 (2 – 15), and Matts score was 2.9 ± 0.2 (2 – 4). Two of the patients with CD were receiving no treatment, and 4 were receiving 5-aminosalicylic acid (5-ASA). Two of the patients with UC were receiving no treatment, and 2 were receiving prednisolone, 6 were receiving oral 5-ASA or sulfasalazine with or without 5-ASA enema. The experimental protocol was reviewed and approved in advance by the ethics committees of Chiba University (Permit number: 697) and the RIKEN Yokohama Institute (Permit number: H17-12).

### Animals

BALB/cA and *Rag1* ^−/−^ mice were obtained from CLEA Japan (Tokyo, Japan). The mice were maintained under specific pathogen-free conditions in RIKEN animal facilities until use in experiments at 8 to 12 weeks old. All animal experiments were approved by the Animal Research Committee of RIKEN Yokohama Research Institute (Permit number: 24-005).

### T cell Preparation

Colonic lamina propria (LP) lymphocytes were prepared as described previously. Briefly, colonic tissues were treated with Hanks’ Balanced Salt Solutions (Wako Pure Chemical Industries) containing 1 mM dithiothreitol and 5 mM EDTA at 37°C for 20 minutes to remove epithelial cells. The tissues were then minced and dissociated with collagenase solution containing 0.5 mg/mL collagenase (Wako Pure Chemical Industries), 1 U/mL dispase (BD Biosciences), 0.5 mg/mL DNase I (Roche Diagnostics), 2% FCS, 100 U/mL penicillin, 100 µg/mL streptomycin, and 12.5 mM HEPES (pH 7.2) in RPMI 1640 medium (Sigma-Aldrich) at 37°C for 30 minutes to obtain single-cell suspensions. After filtering, the single-cell suspensions were washed with 2% FCS/RPMI 1640, and subjected to Percoll gradient separation. Spleen and mesenteric lymph nodes (MLNs) were mechanically disrupted into single-cell suspensions.

### Induction of Colitis by Adoptive Transfer of CD4^+^CD45RB^high^ T cells and by Retransfer of Colitogenic CD4^+^ T cells

Colitis was induced in *Rag1* ^−/−^ mice by adoptive transfer of CD4^+^CD45RB^high^ T cells as described previously [11]. Briefly, CD4^+^ T cells were enriched from splenocytes from BALB/c mice by the MACS system (Miltenyi Biotec) with biotin-conjugated anti-CD4 monoclonal antibody (RM4-5; BD Biosciences) and anti-biotin microbeads (Miltenyi Biotec). Enriched CD4^+^ T cells were labeled with FITC-conjugated anti-mouse CD3ε (145-2C11), APC-conjugated anti-mouse CD25 (PC61), and PE-conjugated anti-mouse CD45RB (16A) (all from BD Biosciences), and CD3ε^+^CD4^+^CD25^−^CD45RB^high^ cells were isolated by cell sorting using FACSAria II flow cytometer (BD Biosciences). The *Rag1* ^−/−^ recipients were each given 1×10^5^ CD4^+^CD45RB^high^ T cells *via* the tail vein and were sacrificed at 8 weeks after transfer. In retransfer experiments, CD3ε^+^CD4^+^CD25^−^CXCR6^−^ and CD3ε^+^CD4^+^CD25^−^CXCR6^+^ cells were isolated from colonic lamina propria of *Rag1* ^−/−^ recipients at 8 weeks after adoptive transfer of CD4^+^CD45RB^high^ T cells by cell sorting using a FACSAria II flow cytometer. CXCL16-Fc fusion protein was used to detect CXCR6-expressing cells. These two populations were retransferred into untreated *Rag1* ^−/−^ recipients.

### Flow Cytometric Analysis

Lymphocytes were incubated with a mouse CXCL16-human IgG Fcγ fusion protein or control human IgG Fcγ, and specific binding was detected with biotinylated anti-human IgG Fcγ (eBioscience) in combination with streptavidin-APC-Cy7 (BD Biosciences). To characterize cell populations, lymphocytes were incubated with FcγR (CD16/CD32)-blocking mAb (93; eBioscience), and further stained with the following mAbs: FITC-conjugated anti-mouse CD62L (MEL14; BD Biosciences); V500-conjugated anti-mouse CD3ε (500A2; BD Biosciences); Pacific Blue-conjugated anti-mouse CD4 (RM4-5; BD Biosciences); PE-conjugated anti-mouse CD27 (LG.3A10; BD Biosciences); PE-Cy7-conjugated anti-mouse CD44 (IM7; eBioscience); FITC-conjugated anti-mouse CD43 (S7; BD Biosciences) and Alexa Fluor 700-conjugated anti-mouse CD127 (A7R34; eBioscience). Flow cytometric analysis was performed for the stained cells using a FACSAria II flow cytometer with DIVA software (BD Biosciences). To analyze intracellular cytokiness, cells were fixed and permeabilized using Cytofix/Cytoperm solution (BD Biosciences) and stained with FITC-conjugated anti-mouse IFN-γ (XMG1.2: eBioscience), PE-conjugated anti-mouse IL-17A (TC11-18H10; BD Biosciences). For transcription factors, Foxp3/transcription factor staining buffer set (eBioscience), respectively. The cells were then and T-bet (4B10; eBioscience), and Alexa Fluor 647-labeled RORγt (Q31-378; BD Biosciences).

### Q-PCR

Total RNA was isolated using an RNeasy Mini Kit (Qiagen), and aliquots of 1 µg of extracted RNA were subjected to reverse transcription (RT) reaction using ReverTra Ace-α (TOYOBO). Real-time PCR analysis was performed to quantify the *Cxcl16* and *Cxcr6* mRNA expression levels using the SYBR Green PCR assay on a Thermal Cycler Dice Realtime System (TAKARA BIO). The expression of the target gene determined by RT-PCR was presented as a ratio, normalized to an endogenous reference (*Gapdh*). The specific primers were: 5′-GGC TTT GGA CCC TTG TCT CTT G-3′ (forward) and 5′-TTG CGC TCA AAG CAG TCC ACT-3′ (reverse) for mouse Cxcl16; 5′-AGA ATT TCT TCC GAC TCC CCG -3′ (forward) and 5′-CAG CTC ATC AAT TCC TGA ACC C-3′ (reverse) for human *CXCL16*; and 5′-GGA CAT TGG TTG CCT CCC TTA-3′ (forward) and 5′-AAA CAA AGC CTG CCT CAC CAC-3′ (reverse) for human *CXCR6*.

### Immunohistochemistry

For immunohistochemical analysis of human CXCL16 and CXCR6, the biopsy samples were fixed in 1% zinc sulfate/4% formalin (Richard-Allan Scientific). Sections of human mucosa 5-µm thick were deparaffinized, rehydrated, and treated with 0.3% H_2_O_2_ in PBS for 20 min at room temperature to block endogenous peroxidase activity. The sections were incubated with 5% bovine serum albumin in PBS for 30 min at room temperature and then with goat anti-human CXCL16 polyclonal Ab (R&D Systems), mouse anti-human CXCR6 monoclonal Ab (R&D Systems), or an identical concentration of control goat or mouse IgG, overnight at 4°C. The binding of primary Ab was detected with biotinylated donkey anti-goat or mouse IgG (DAKO) followed by streptavidin-horseradish peroxidase (ABC Elite; Vector Laboratories), visualized with 3,3′-diaminobenzidine (DAKO), and counterstained with hematoxylin (DAKO). Immunostaining of mouse CXCL16 was described previously [12].

### Microarray Data Collection and Analysis

Total RNA were prepared from CXCR6^+^CD4^+^ T cells and CXCR6^−^CD4^+^ T cells in the colon of colitic mice, and CD45RB^high^CD4^+^ naïve T cells in the spleen of BALB/c mice using a RNeasy Plus Mini kit (Qiagen). RNA was amplified and hybridized on the GeneChip Mouse Genome 430 2.0 Array (Affymetrix), according to the manufacture’s procedure. Expression values were determined with Gene Spring version 11.5 (Tomy Digital Biology). The data has also been submitted to GEO database (accession# GSE45881).

### Statistical Analysis

Differences between two groups were analyzed by the Student’s *t* test, unless otherwise specifically noted. When variances were unequal, the data were analyzed by Mann-Whitney U test. In all analyses, *P* < 0.05 was taken to indicate significance.

## Results

### Expression of CXCL16 and CXCR6 is Upregulated in the Inflamed Colon of CD Patients and a Mouse Model of CD Colitis

**Figure 1:**
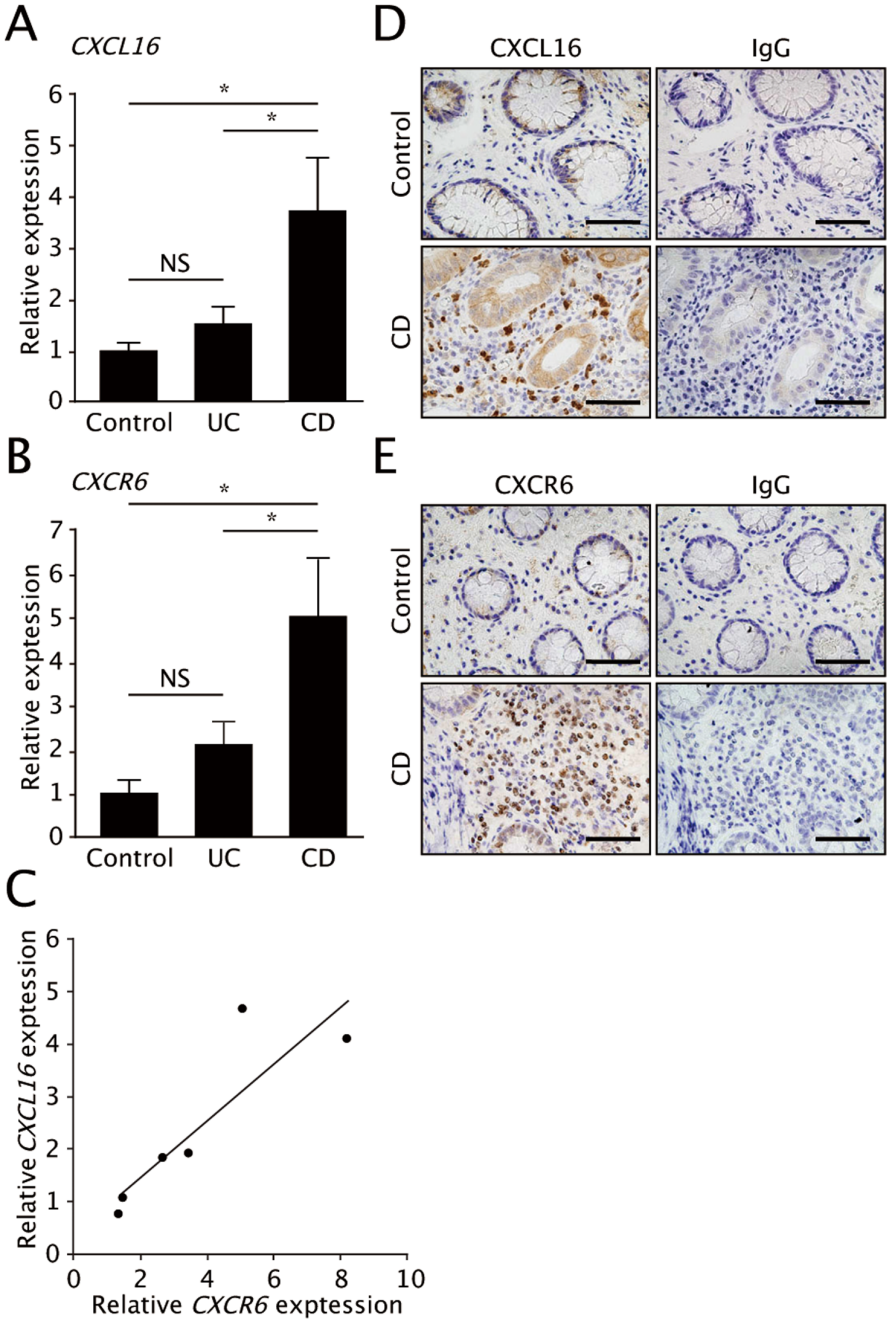
CXCL16 and CXCR6 are upregulated in colonic mucosa of CD patients. (**A, B**) mRNA expression of *CXCL16* (**A**) and *CXCR6* (**B**) was evaluated by Q-PCR. The expression of *CXCL16* and *CXCR6* is higher in colonic mucosa of patients with Crohn’s disease (CD) compared with patients with ulcerative colitis (UC) and healthy controls. Data were normalized to expression of *GAPDH* mRNA. (*n = *5–10; mean and s.e.m.). *, *P* < 0.05. (**C**) Correlation between *CXCL16* and *CXCR6* mRNA expression in the colonic mucosa of CD patients. Statistical analysis was performed by Spearman’s correlation; correlation coefficient = 0.76, *P* = 0.024. (**D, E**) Immunohistochemistry of CXCL16 (**D**) and CXCL16 (**E**) was performed on colonic mucosa of patients with CD and healthy controls. CXCL16 positive staining was observed on epithelial cells and a subset of colonic LP cells in CD patients (**D**). CXCR6 was strongly expressed by small round cells in CD mucosa (**E**). Scale bars, 50 µm, IgG indicates a control antibody. Representative photomicrographs obtained from the analysis of five or six specimens per group are shown.

To gain insight into the pathological relevance of the CXCL16-CXCR6 system, we first investigated the expression of these molecules in inflamed colonic mucosa of patients with IBD. Quantitative PCR (Q-PCR) analysis showed that the expression level of the genes encoding *CXCL16* and *CXCR6* was significantly increased in the mucosa of CD patients compared to healthy subjects and UC patients (Fig. 1A, 1B). The upregulation of CXCL16 was also confirmed at the protein level by Western blot analysis (data not shown). Furthermore, there was a significant correlation between *CXCL16* and *CXCR6* expression in CD patients (Fig. 1C). By contrast, there were no statistically significant differences in the expression of these genes between UC patients and healthy subjects (Fig. 1A, 1B). Immunohistochemical studies confirmed that CXCL16 was highly expressed by a fraction of LP cells, most likely myeloid cells such as dendritic cells and/or macrophages because of their polymorphic cell shape with relatively large cytoplasm, in the inflamed mucosa of CD patients (Fig. 1D). In addition, colonic epithelium exhibited moderate CXCL16 expression. On the other hand, CXCR6 expression was observed on small round cells with the appearance of infiltrating lymphocytes in the colon of CD patients but not healthy controls (Fig. 1E).

**Figure 2:**
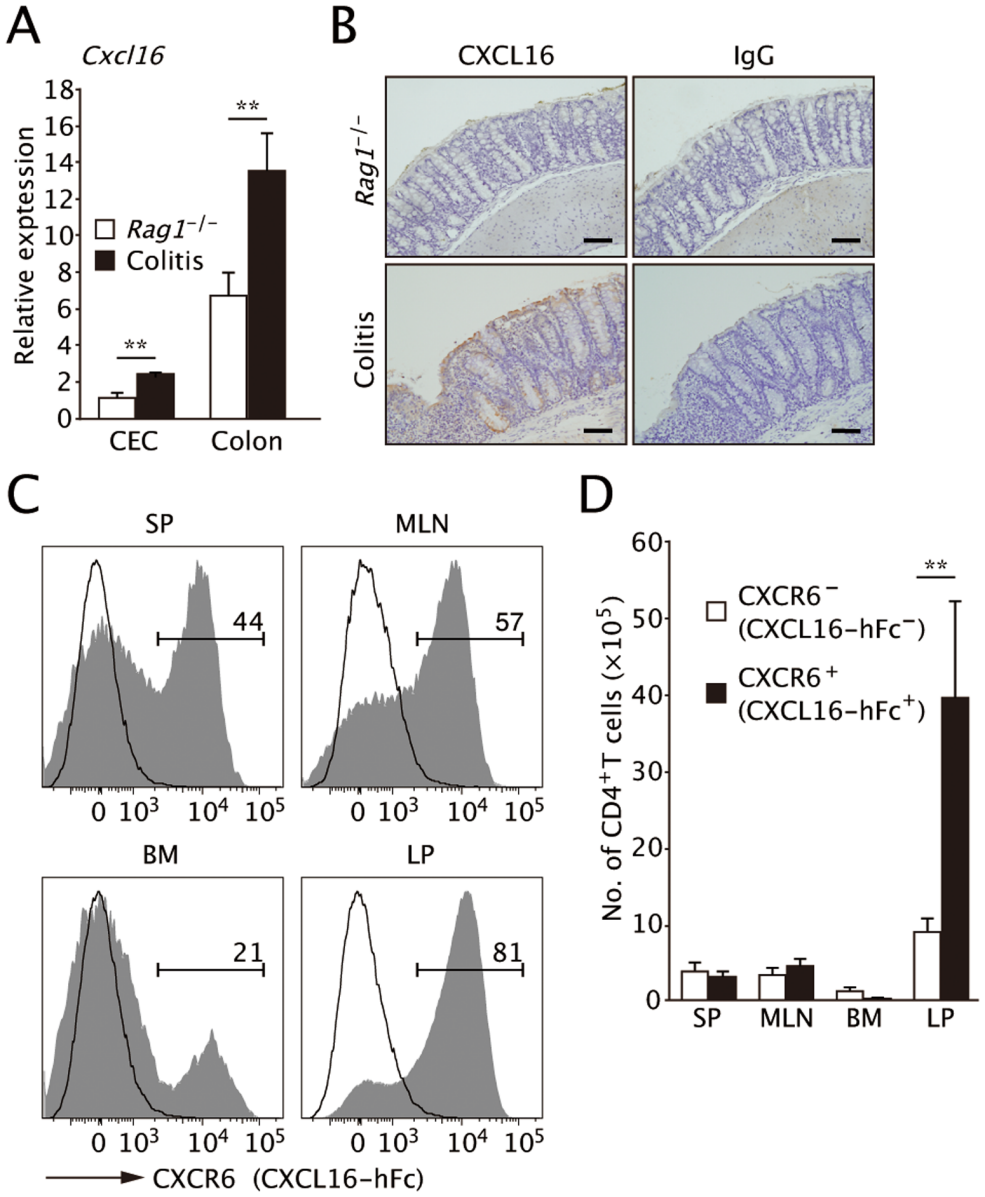
Expression of CXCL16 increased in the inflamed mucosa of CD45RB^high^ transfer colitis. (**A**) *Cxcl16* mRNA levels in colonic epithelium (CEC) and distal colon tissues were analyzed by Q-PCR 8 weeks after transfer. The expression of *Cxcl16* mRNA increased in both epithelium and colon tissues of the transfer model compared with healthy *Rag1^−/−^* mice. Data were normalized to expression of *Gapdh* mRNA. (*n = *5; mean and s.d.). **, *P* < 0.01. (**B**) CXCL16 immunostaining of the distal colon in colitic and healthy *Rag1^−/−^* mice. Scale bars, 100 µm. Data are representative of two independent experiments. (**C**) CXCR6 expression on CD4^+^ T cells was analyzed by flow cytometry using a mouse CXCL16-human IgG-Fc fusion protein or control human IgG-Fcγ at 8-week post transfer. CXCR6 was expressed at high levels by the majority of colonic LP CD4^+^ T cells in the colitic mice, by about half of the SP and MLN CD4^+^ T cells, and by ∼20% of BM cells. (**D**) Absolute numbers of CXCR6^+^ and CXCR6^−^ CD4^+^ T cells in each of tissues were calculated based on the flow cytometric analysis described in (**C**). Data are representative of three independent experiments (mean and s.d.). **, *P* < 0.01.

We further examined CXCL16 expression in a well-characterized mouse model of CD colitis induced by adoptive transfer with CD45RB^high^ naïve CD4^+^ T cells into immunodeficient *Rag1^−/−^* mice. Similar to the situation in CD patients, *Cxcl16* mRNA levels were upregulated in colonic epithelial cells (CEC) and whole colon tissue from the colitic mice compared to healthy *Rag1^−/−^* mice (Fig. 2A). Immunohistochemical staining also demonstrated that CXCL16 protein was expressed by CECs, endothelial cells, and a subpopulation of immune cells in the inflamed colon tissue (Fig. 2B).

We subsequently analyzed distribution of CXCR6-expressing cells using a mouse CXCL16-human IgG-Fc fusion protein [13]. Although splenic CD4^+^CD45RB^high^ naïve T cells before the adoptive transfer lacked CXCR6 expression, 10.9% of CD4^+^CD45RB^low^ T cells expressed CXCR6 at intermediate levels (Fig. S1). Those CD4^+^CD45RB^low^CXCR6^int^ T cells had a CD44^+^CD62L^−^ effector memory phenotype. On the other hand, CD4^+^CD45RB^low^CXCR6^−^ T cells were a heterogeneous population that included cells of both CD44^−^CD62L^+^ naïve and CD44^+^CD62L^−^ effector memory phenotypes (Fig. S1C, S1D).

Most CD4^+^ T cells in colonic LP strongly expressed CXCR6 in colitis model mice at 8 weeks after adoptive transfer (Fig. 2C, 2D). Splenic and MLN CD4^+^ T cells expressed CXCR6 to a lesser extent, and only a small portion of BM CD4^+^ T were are positive for CXCR6 (Fig. 2C, 2D).

### CXCR6^−^ and CXCR^+^CD4^+^ T cells Subsets in the Inflamed Colon both Contain Effector and Effector-memory Cells

**Figure 3:**
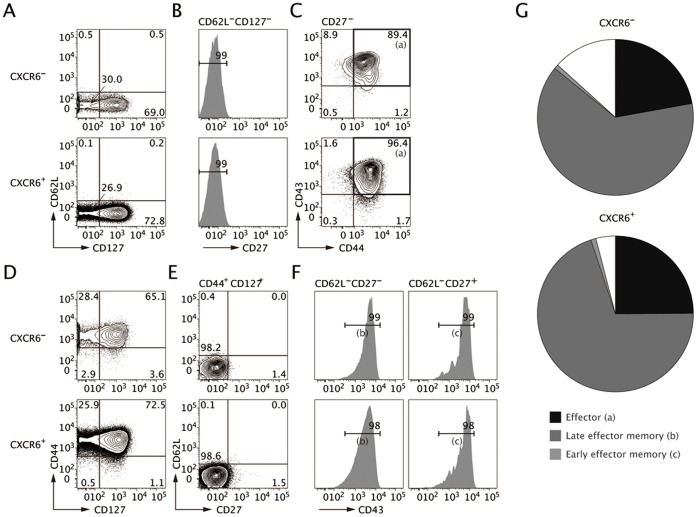
CXCR6 is expressed both by the effector and effector memory CD4^+^ T cells in the inflamed colon. LP CXCR6^−^ and CXCR6^+^ cells were analyzed for expression of activation and memory markers at week 8 post-transfer of naïve CD4^+^ T cells. (**A**–**C**) CD4^+^ T cells were gated as CD127^−^CD62L^−^CD27^−^CD43^+^CD44^+^ to measure the proportion of effector T cells (a). (**D**–**F**) The effector memory population (CD44^+^CD127^+^) was subdivided using CD62L and CD27 to measure early effector memory cells (CD62L^−^CD27^+^CD43^+^, b) and late effector memory cells (CD62L^−^CD27^−^CD43^+^, c). Data are representative of three independent experiments. (**G**) The relative percentages of effector, early effector memory and late effector memory in each subset are shown in a pie chart.

To further characterize CXCR6-expressing CD4^+^ T cells in the inflamed colon, we analyzed their expression of the activation and the memory markers: CD27, CD43, CD44, CD62L, and CD127. Colonic LP CXCR6^−^ and CXCR6^+^ subsets equally contained effector cells of the phenotype CD27^−^CD44^+^CD43^+^CD62L^−^CD127^−^ (Fig. 3A–C). Furthermore, there was no difference in the proportion of CD44^+^CD127^+^ effector-memory cells between the two groups (Fig. 3D). CD44^+^CD127^+^ effector-memory CD4^+^ T cell population can be subdivided into late and early effector-memory populations based on CD27 and CD62 expression, CD27^+^CD62L^−^ and CD27^−^CD62L^−^ cells representing early and late effector-memory populations, respectively [14], [15]. Colonic LP CXCR6^−^ and CXCR6^+^ subsets predominantly contain late rather than early effector memory cells (Fig. 3D–F). These data together demonstrate that the two subsets are composed of an almost identical proportion of effector and late effector-memory cell types (Fig. 3G). However, this was not the case in the spleen, where the SP CXCR6^−^ subset contained fewer effector and late effector memory cells but more early effector memory cells as well as central memory cells (CD44^+^CD127^+^CD27^+^CD62L^+^CD43^+^) compared with the CXCR6^+^ subset. MLN cells also showed a similar phenotype (Fig. S2). Asequential pathway of CD4^+^ T-cell differentiation, from central memory to early effector-memory and then late effector-memory cells has been proposed based on surface marker expression and the length of telomeres [15]–[17]. During this process, a high antigen load would enhance generation of late effector-memory cells. These observations suggest that the CXCR6-expressing CD4^+^ T cells may be more antigen-experienced and highly differentiated than the CXCR6^−^ subset in SP and MLN.

### CXCR6^+^CD4^+^ T cells are Responsible for the Production of Inflammatory Cytokines

**Figure 4:**
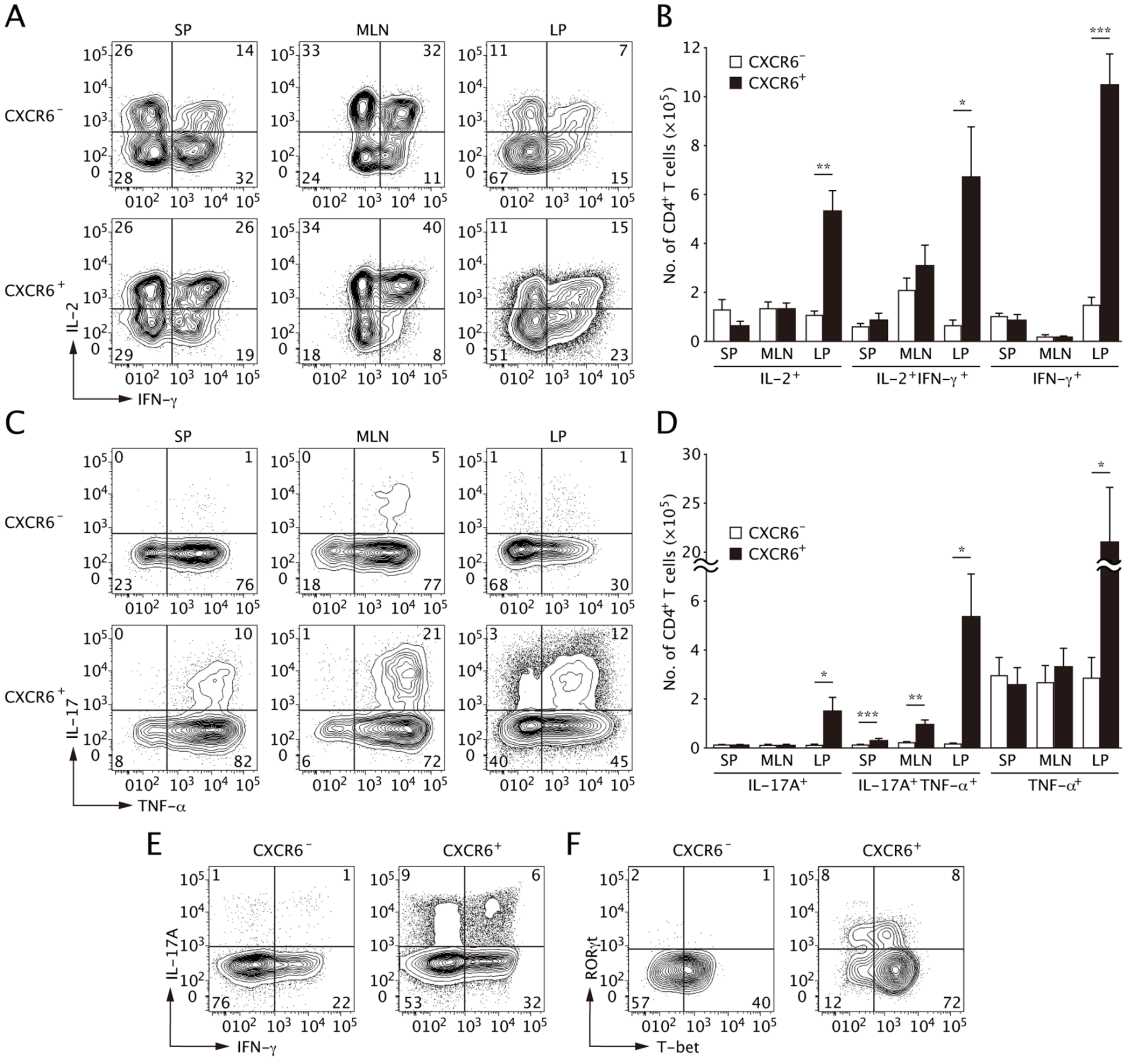
CXCR6 expression is related to the production of Th1 and Th17 cytokines. Intracellular staining for cytokine and transcription factors in CD4^+^ T cells was performed 8 weeks after naïve CD4^+^ T-cell transfer. (**A, B**) The frequency of IL-2^+^ cells and IFN-γ^+^ cells was analyzed in the CXCR6^−^ (upper) and CXCR6^+^ (lower) subsets (**A**) and the absolute number of T cells were graphed on the basis of the flow cytometric analysis (**B**). (**C, D**) The frequency of IL-17A^+^ cells and TNF-α^+^ cells was analyzed in CXCR6^−^ (upper) and CXCR6^+^ (lower) subset (**C**) and the numbers were graphed on the basis of flow cytometric analysis (**D**). (**E**) The frequency of IFN-γ^+^ cells and IL-17A^+^ cells was analyzed. (**F**) The frequency of T-bet^+^ cells and RORγt^+^ cells was analyzed. All data are representative from four independent experiments (mean and s.d.). *, *P* < 0.05 **, *P* < 0.01 ***, *P* < 0.001.

In order to determine whether CXCR6 expression on CD4^+^ T cells relates to an effector function, we performed intracellular cytokine staining on SP, MLN and colonic LP cells from colitis model mice at 8 week post-transfer. In the colonic LP, the proportions of IFN-γ^+^ cells and IL-2^+^IFN-γ^+^ cells were higher in the CXCR6^+^ subset than in the CXCR6^−^ subset (Fig. 4A). Considering that the majority of colonic CD4^+^ T cells expressed CXCR6 (Fig. 2C), the source of IL-2 and IFN-γ in the inflamed colon was mainly the CXCR6^+^ subset (Fig. 4B). An *in vitro* Th1 differentiation assay with CFSE-labeled CD4^+^ naïve T cells showed that CFSE^−^CXCR6^+^ cells preferentially produced IFN-γ compared with CFSE^+^CXCR6^+^ cells and CXCR6^−^ cells (Fig. S3). Additionally, IL-17A-producing cells (IL-17A^+^ and IL-17A^+^TNF-α^+^) were predominantly a CXCR6^+^ subset (Fig. 4C, 4D). Immunohistochemical studies also confirmed expression of IL-17A by CXCR6^+^ cells in colonic mucosa of CD patients (Fig. S4). Thus, even though surface markers other than CXCR6 were quite similar between the CXCR6^+^ and CXCR6^−^ subsets, the cytokine production profile clearly distinguished the two populations. Together, the highly expanded CXCR6^+^ subset seems to mediate chronic inflammatory responses in the effector site by producing abundant Th1 and Th17 effector cytokines.

In SP and MLN, the CXCR6^+^ CD4^+^ T subset contained a greater proportion of IL-2^+^IFN-γ^+^ double-producing cells (Fig. 4A, 4B). Furthermore, the proportion and number of IL-17A^+^TNF-α^+^ double-producing cells was much higher in the CXCR6^+^ subset than in the CXCR6^−^ subset (Fig. 4C, 4D). Of note, more than half of the IL-17A-producing CXCR6^+^ cells co-expressed IFN-γ (Fig. 4E). Intracellular staining of T-bet and RORγt also supported the observation that CXCR6^+^ subset is composed mainly of Th1 (T-bet^+^) and Th17 (RORγ^+^) cells (Fig. 4F). Consistent with the co-expression of IL-17A and IFN-γ, the half of RORγt^+^CXCR6^+^ subset co-expressed T-bet.

### CXCR6 Expression on CD4^+^ T cells is not Required for the Accumulation of Colitogenic T cells

**Figure 5:**
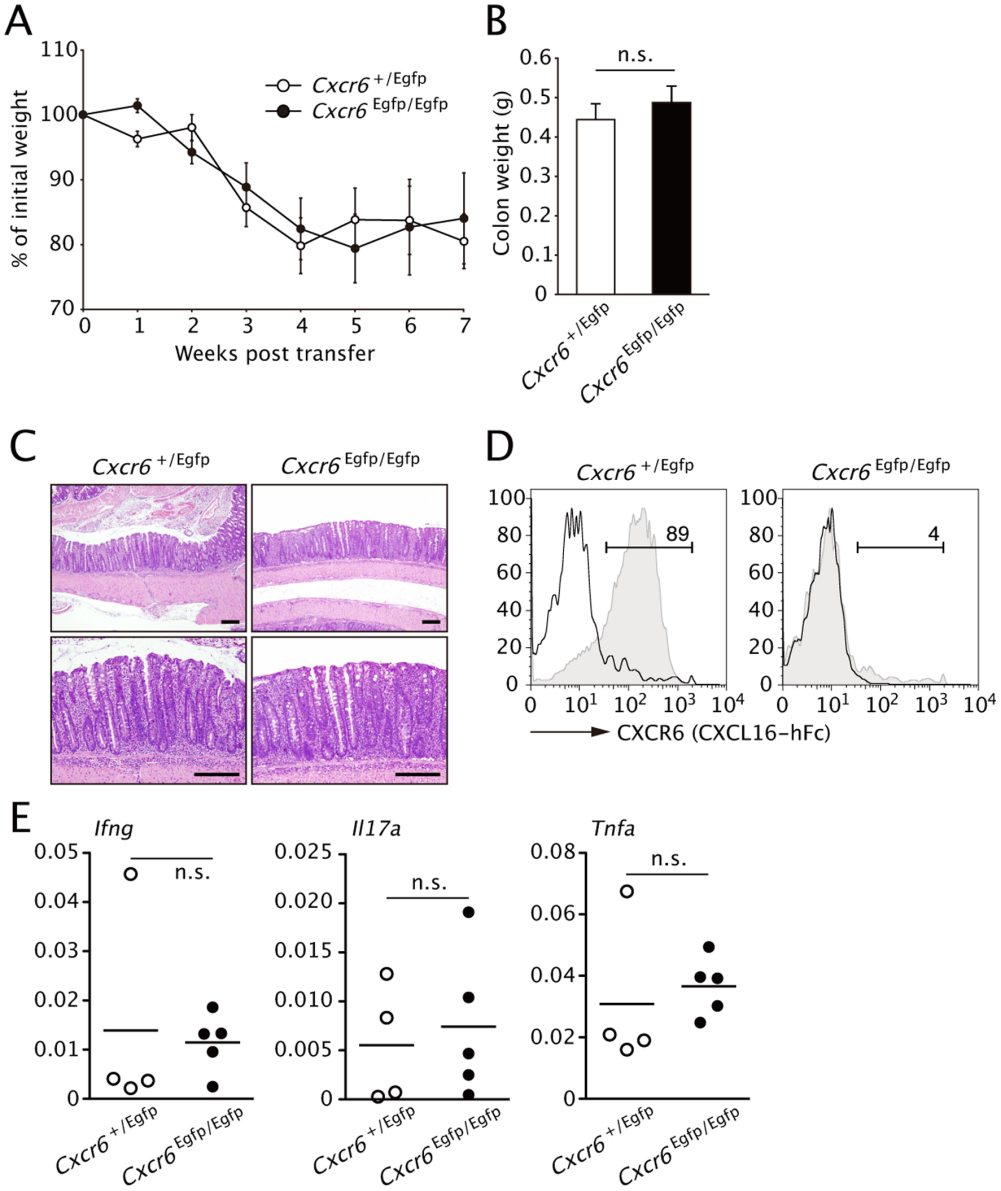
CXCR6 expression is not required for development of transfer colitis. (**A**) Body weight of *Rag1^−/−^* recipients of i.v. injected purified CD45RB^high^CD4^+^ T cells form *Cxcr6* ^+/Egfp^ or *Cxcr6* ^Egfp/Egfp^ (CXCR6-deficient) mice on day 0, presented as percent of original weight. (**B**) Colon weight of the mice in (**A**) on week 7. Data are representative of two independent experiments (mean and s.d.). (**C**) Histology of colon tissues from the mice in **B**. (**D**) CXCR6 expression by LP CD4^+^ T cells was analyzed by flow cytometry using CXCL16-hFc at 7-week post transfer. (**E**) Expression levels of indicated cytokines in distal colon were analyzed by Q-PCR at 7 weeks after the transfer. Data were normalized to expression of *Gapdh*. (*n = *4 or 5; mean and s.d.).

Given the upregulation of CXCL16 in the inflamed colon as well as the active role of CD4^+^CXCR6^+^ T cells in cytokine production, the CXCL16-CXCR6 system may be important for the development and persistence of the colonic inflammation. This possibility was directly assessed by the adoptive transfer of CD45RB^high^ naïve CD4^+^ cells isolated from CXCR6-deficient mice (*Cxcr6^Egfp/Egfp^* mice) into *Rag1^−/−^* recipients. CXCR6-EGFP heterozygous mice (*Cxcr6^+/Egfp^*) were used as positive control donor. The adoptive transfer of the CXCR6-deficient T cells induced a wasting disease associated with increased colon weight to a similar extent the control cells (Fig. 5A, 5B). Histopathological analysis of the distal colon confirmed the development of chronic inflammation in both groups (Fig. 5C). The absence of CXCR6 expression by LP CD4^+^ T cells isolated from recipients of *Cxcr6^Egfp/Egfp^* CD4^+^ cells was confirmed by flow cytometry (Fig. 5D). This observation raises the possibility that colitogenic CD4^+^ T cells may redundantly express other chemokine receptors to migrate into colonic lamina propria. In gene expression profiling, colitogenic CD4^+^ T cells displayed upregulation of 8 chemokine receptors including CXCR6 (Table S1).

We also examined whether CXCL16-CXCR6 axis is involved in the signal transduction for proinflammatory cytokine production. The expression levels of effector cytokine transcripts in the inflamed colon tissues were comparable regardless of CXCR6 expression on the adoptively transferred CD4^+^ T cells (Fig. 5E). Consistently, the production of IL-2 and IFN-γ by CXCR6^+^CD4^+^ T cells were comparable when the cells were cultured in Th1-conditioned medium with and without supplementation of recombinant mouse CXCL16 or CXCL16-Fc. (Fig. S5). Taken together, these data imply that the CXCL16-CXCR6 system appears to be dispensable for the migration and function of colitogenic T cells.

### CXCR6^−^CD4^+^ T cells but not CXCR6^+^CD4^+^ T cells can Transfer Colitis

**Figure 6:**
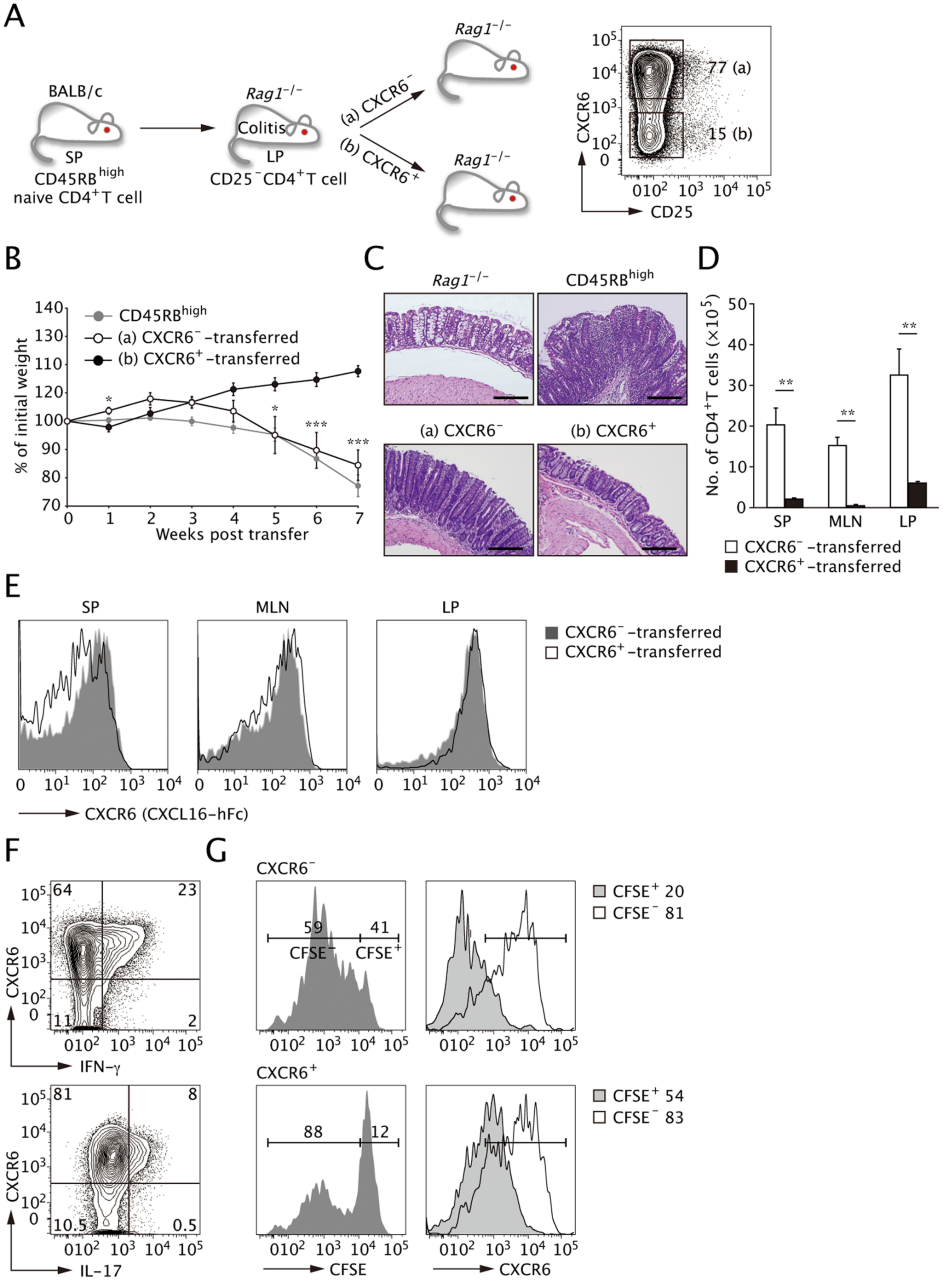
LP CXCR6^−^ but not CXCR6^+^ cells can transfer wasting and colitis upon retransfer into *Rag1^−/−^* recipients. (**A**) Schematic transfer protocol. An equal number of CD25^−^CXCR6^−^ cells or CD25^−^CXCR6^+^CD4^+^ T cell isolated from colon LP of colitic mice was retransferred into *Rag1^−/−^* mice. (**B**) Time course of changes in body weight after retransfer of the two subsets or transfer of CD45RB^high^ naïve T cells. CXCR6^–^transferred *Rag1^−/−^* mice manifested progressive body weight loss to a similar extent to CD45RB^high^-transferred *Rag1^−/−^* mice, whereas the cohort that received CXCR6^+^cells did not show wasting. Data are expressed as the mean and s.e.m of two independent experiments. *, *P* < 0.05 ***, *P* < 0.001 versus CXCR6^+^-transferred animals. (**C**) Histopathological analysis of distal colon tissue at 8 weeks post transfer. The transfer of CXCR6^−^CD4^+^ T cells alone induced intestinal inflammation. Scale bars, 50 µm. (**D**) Number of CD4^+^ T cells in each group were calculated based on the flow cytometric analysis. (**E**) CXCR6 expression on CD4^+^ T cells in each group was analyzed by flow cytometry using CXCL16-hFc at 8-week post transfer. CXCR6^−^CD4^+^ T cells express CXCR6 upon retransfer. (**F**) Intracellular cytokine staining was performed in LP CD4^+^ T cells in recipients of CXCR6^−^CD4^+^ T cells at 8 weeks after transfer. The CXCR6^−^ subset produced IFN-γ and IL-17A coincident with the expression of CXCR6 upon retransfer. (**G**) LP CXCR6^−^ and CXCR6^+^ subsets were purified from CD45RB^high^-transferred *Rag1^−/−^* mice at 8-week post transfer, labeled with CFSE and cultured with LP MHC class II^+^CD11c^+^ cells at a 5∶1 ratio in the presence of 5 µg/ml anti-CD3ε Abs and 20 ng/ml IL-23 for 3 days. Proliferation was measured by CFSE dilution. Proliferation by CXCR6^−^ and CXCR6^+^ cells is depicted in the right columns. All data are representative from four independent experiments (mean and s.d.).

To further assess the roles of CXCR6^+^ and CXCR6^−^ T cells in the pathogenesis of colitis, we next performed adoptive retransfer of LP CXCR6^+^ and CXCR6^−^ CD4^+^ T cells recovered from the inflamed colon. To avoid carry over of regulatory T cells into the recipient mice, we eliminated the CD25^high^ population from the donor cells (Fig. 6A). Unexpectedly, *Rag1^−/−^* recipients of the CXCR6^−^ subset exhibited progressive body weight loss with clinical symptoms of colitis to a similar extent as the recipients of splenic CD45RB^high^ naïve CD4^+^ T cells. On the other hand, the recipients of the CXCR6^+^ subset exhibited mild body weight loss at 1 week post-transfer; however, they recovered quickly and remained healthy for the 8-week duration of the study (Fig. 6B). The colon/body weight ratio of the recipients of the CXCR6^−^ subset was significantly higher than the CXCR6^+^ subset at 8 weeks (data not shown). Histopathological analysis of the distal colon tissues from the two groups indicated that the transfer of the CXCR6^−^ but not CXCR6^+^ subset induced chronic inflammation similar to that observed in CD45RB^high^-transferred *Rag1^−/−^* recipients (Fig. 6C). These observations indicate that only the CXCR6^−^ subset retains the ability to transfer the disease. *Rag1^−/−^* recipients reconstituted with CXCR6^+^ cells contained a limited number of CD4^+^ T cells in SP, MLN, and colon LP that retained CXCR6 expression (Fig. 6D, 6E). In sharp contrast, the CXCR6^−^ cells retransferred into *Rag1^−/−^* recipients vigorously expanded and the majority of them upregulated CXCR6 expression. The conversion of retransferred CXCR6^−^ into CXCR6^+^ cells was most prominent in LP among the tissues tested (Fig. 6E). The CD4^+^ T cells that acquired CXCR6 expression predominantly produced effector cytokines such as IFN-γ and IL-17A (Fig. 6F), suggesting that CXCR6^+^ cells generated *in situ* from CXCR6^−^ cells mediate the colonic inflammation.

The fact that recipients of retransferred CXCR6^+^ cells failed to develop the disease raised the possibility that this could be a population of terminally differentiated effector cells with little proliferative activity. Indeed, the CXCR6^+^ subset was less proliferative compared to the CXCR6^−^ counterpart upon *in vitro* restimulation (Fig. 6G). Collectively, these observations indicate that the CXCR6^−^ cells actively expand, giving rise to CXC6^+^ effector T cells that are responsible for the persistence of the chronic colitis.

## Discussion

The CXCL16-CXCR6 system is involved in the pathogenesis of several inflammatory disorders including rheumatoid arthritis and chronic liver inflammation by recruiting CD4^+^ and CD8^+^ T cells [18]–[21]. More recently, two groups have demonstrated that CXCL16 is upregulated in the inflamed colonic tissue and serum of CD patients [3], [5]. We here demonstrate that CXCR6^+^ lymphocytes accumulated within the inflammatory lesions in CD patients. In accordance with this observation, the majority of LP CD4^+^ T cells expressed CXCR6 in the mouse model of colitis induced by adoptive transfer of CD4^+^CD45RB^high^ T cells. The frequency of CXCR6^+^CD4^+^ T cells was much higher in the colon than in the SP and MLN of these mice. Both the major CXCR6^+^ and minor CXCR6^−^ CD4^+^ T-cell subsets were mainly composed of effector (CD62L^−^CD127^−^CD27^−^CD44^+^CD43^+^) and late effector memory (CD62L^−^CD127^+^CD27^−^CD44^+^) cells [14], [15]. Nevertheless, the two subsets were functionally distinct; only the LP CXCR6^+^ subset recovered from the inflamed tissue abundantly produced inflammatory effector cytokines such as TNF-α, IFN-γ and IL-17A. Retransfer of the LP CXCR6^+^ cells, however, failed to induce colitis. This unexpected result is ascribed to the lack of proliferative activity in the CXCR6^+^ subset, suggesting that this cell population consists of the short-lived, finally differentiated effector cells. By contrast, much fewer cells in the CXCR6^−^ subset produce effector cytokines but the cells nonetheless retain the ability to proliferate and differentiate into CXCR6^+^ effector cells *in situ* after retransfer. We and others have reported that CD4^+^ T cells upregulate CXCR6 upon stimulation with anti-CD3ε and CD28 mAbs [12], [20], [22]. Therefore, it is plausible that retransferred CXCR6^−^ cells undergo homeostatic proliferation in the *Rag1* ^−/−^ host and some of them migrate into the gut, where they are activated by, most likely, microbial antigens, and fully differentiate into cytokine-producing CXCR6^+^ effector cells. Studies of both CD8^+^ and CD4^+^ T-cell memory in chronic viral or parasitic infection have correlated the memory phenotype with the level and duration of antigen stimulation; a low level chronic infection would lead to maintenance of early effector memory cells, whereas a high level infection would enhance late effector-memory cells [15], [23]. In addition to such a parallel differentiation pathway, a linear pathway of CD4^+^ T-cell differentiation has been proposed; namely, early effector memory cells could differentiate into late effector memory cells, and eventually to effector cells [14], [15], [23]. In the CD4^+^CD45RB^high^-transfer model, CD4^+^ T cells should be exposed to a large amount of antigens derived from the commensal microbiota in the gut. It is thus reasonable that the majority of colonic CD4^+^ T cells in this model indeed display late effector-memory and effector phenotypes. The differentiation from CXCR6^−^ to CXCR6^+^ cells must be an ongoing process during the course of chronic inflammation, given the abundance of CXCR6^+^ cells in the inflamed colon despite their being short-lived. Therefore, the CXCR6^−^ subset is most likely responsible for the persistence of chronic inflammation in the gut.

Previous studies indicated that CXCL16 is induced on the surface of myeloid cells such as macrophages and dendritic cells upon activation [18], [24], and is also constitutively expressed by follicle-associated epithelium, which is immunologically activated by stimuli from the underlying lymphoid follicles in the Peyer’s patch [12], [25]. Inflammatory cytokines such as TNF-α and INF-γ synergize to induce CXCL16 in the intestinal epithelial cells [26]. In accordance with these reports, we also observed that myeloid and epithelial cells express this chemokine in the inflamed colon where TNF-α and INF-γ are abundantly expressed. Although the CXCL16-CXCR6 system seems to be dispensable for the development of chronic colitis in the CD4^+^CD45RB^high^-transferred model as described here, another group has suggested that gene-targeting deletion or neutralization of CXCL16 ameliorates colonic inflammation in experimental colitis induced by dextran sulfate sodium or trinitrobenzene sulfonic acid [5]. This response is at least partly due to downregulation of the inflammatory response in CXCL16-deficient macrophages [5]. These data imply that the CXCL16-CXCR6 system plays a role in certain aspects of the inflammatory response in the gut. Similarly, colitogenic invariant natural killer (iNKT) cells also accumulate in colonic LP in a CXCL16-dependent manner and increase morbidity in oxazolone-induced experimental colitis under germ-free conditions [27]. However, in the CD45RB^high^ T-cell*-*transfer colitis model, we show that CXCR6-deficient CD4^+^ T cells retain the ability to induce wasting and colitis. The numbers of CD4^+^ T cells infiltrating into the colonic tissue were comparable in mice transferred with CXCR6-deficient or control CD4^+^CD45RB^high^ cells (data not shown). This result indicates that CXCR6 is dispensable for recruiting CD4^+^ T cells to the colonic LP. CXCR6 deficiency seems to be compensated by other chemokine receptors in this experimental colitis model. Indeed, multiple chemokine receptors are reported to recruit colitigenic CD4^+^ T cells into the site of inflammation in CD patients [9], [10].

Previous studies have linked CXCR6 expression by CD4^+^ and CD8^+^ T cells to IFN-γ but not IL-4 expression in graft-versus-host-induced hepatitis and rheumatoid arthritis [20], [28]. Our data also demonstrate that the frequency of IFN-γ-producing cells is higher in the CXCR6^+^CD4^+^ subset compared to its CXCR6^−^ counterpart in the inflamed colon. However, the most striking difference was observed in the IL-17A expression profiles. Colonic CXCR6^+^ cells preferentially expressed IL-17A, whereas very few CXCR6^−^ cells produced this cytokine. This observation raises the possibility that the CXCL16-CXCR6 axis may play a significant role in the recruitment of Th17 cells to the colonic mucosa. The CD4^+^CD45RB^high^ cell-induced colitis is mainly mediated by a Th1-dominant inflammatory response [29] and contribution of the Th17-response in this model is relatively minor. Therefore, CXCR6 could well be a functional marker for the Th17-type effector, but that CXCR6 deficiency would not affect the development of colitis in a Th1-dominant model such as the CD4^+^CD45RB^high^ cell-transferred model. Further studies using a Th17-dependent colitis model will be required to address this issue. Because CXCR6^−^CD4^+^ T cells are responsible for recurrent inflammatory responses, this cell population could be a potential therapeutic target for the chronic inflammation in CD.

## Supporting Information

Figure S1 **CD45RB^low^CD4 T^+^ cells express CXCR6.** (**A, B**) Splenic CD4^+^ T cells were recovered from BALB/c mice, divided into CD45RB^high^ naïve cells and CD45RB^low^ cells (**A**), and the expression of CXCR6 was analyzed using CXCL16-hIgG Fcg fusion protein (CXCL16-hFc). Solid line, hIgG-Fcγ (**B**). (**C, D**) The expressions of CD44, CD62L and CD25 were compared among CD45RB^high^ naïve, CD45RB^low^CXCR6^−^ and CD45RB^low^CXCR6^+^ CD4^+^ T cells. Data are representative of three independent experiments.
(PPTX)

Figure S2 **SP and MLN CXCR6** ^−^ **and CXCR6^+^CD4^+^ T-cell subsets were analyzed for expression of activation and memory markers on week 8 post-transfer of naïve CD4^+^ T cells.** (A–C) The each subset was gated into CD127^−^CD62L^−^CD27^−^CD43^+^CD44^+^ to measure the proportion of effector T cells (a). (D–G) Memory population (CD44^+^CD127^+^) was subdivided using CD62L, CD27 and CD43 to measure late effector memory cells (CD62L^−^CD27^−^CD43^+^, b) early effector memory cells (CD62L^−^CD27^+^CD43^+^, C) and central memory cells (CD62L^+^CD27^+^, d). Data are representative of three independent experiments. (H) The relative percentages of effector, early effector memory and late effector memory cells in each subset are shown in a pie chart based on (A–G).
(PPTX)

Figure S3 **Well-proliferated CD4^+^ T cells express CXCR6, and their CXCR6 expression is correlated with IFN-g and IL-2 productions.** CFSE-labeled naïve CD4^+^ T cells were differentiated under Th1 condition. On the 6 days of culture, the CXCR6 expression and cytokine production were determined by flow cytometry.
(PPTX)

Figure S4 **CXCR6^+^ cells express IL-17A in CD colitis.** Immunohistochemistry of CXCR6 (left) and IL-17A (right) was performed on serial paraffin sections of colonic mucosa from patients with CD.
(PPTX)

Figure S5 **CXCL16 stimulation does not enhance effector cytokine production.** Naïve CD4^+^ T cells were differentiated under Th1 condition with or without soluble CXCL16 or plate-bound CXCL16-human IgG-Fc fusion protein (CXCL16-hFc). Human IgG-Fc (hFc) was used as a control for CXCL16-Fc. After 6 days of culture, cytokine production in CXCR6^+^ subset was examined by flow cytometry.
(PPTX)

Table S1 (PPTX)
