# Supplementary material for: Distinct Roles for CXCR6+ and CXCR6− CD4+ T Cells in the Pathogenesis of Chronic Colitis
Source: PLoS One. 2013 Jun 19;8(6):e65488. doi: 10.1371/journal.pone.0065488 (PMC3686755; doi:10.1371/journal.pone.0065488)
Supplement: Figure S1 — CD45RBlowCD4 T+ cells express CXCR6. (A, B) Splenic CD4+ T cells were recovered from BALB/c mice, divided into CD45RBhigh naïve cells and CD45RBlow cells (A), and the expression of CXCR6 was analyzed using CXCL16-hIgG Fcg fusion protein (CXCL16-hFc). Solid line, hIgG-Fcγ (B). (C, D) The expressions of CD44, CD62L and CD25 were compared among CD45RBhigh naïve, CD45RBlowCXCR6− and CD45RBlowCXCR6+ CD4+ T cells. Data are representative of three independent experiments. (PPTX) [file pone.0065488.s001.pptx]

## Slide 1
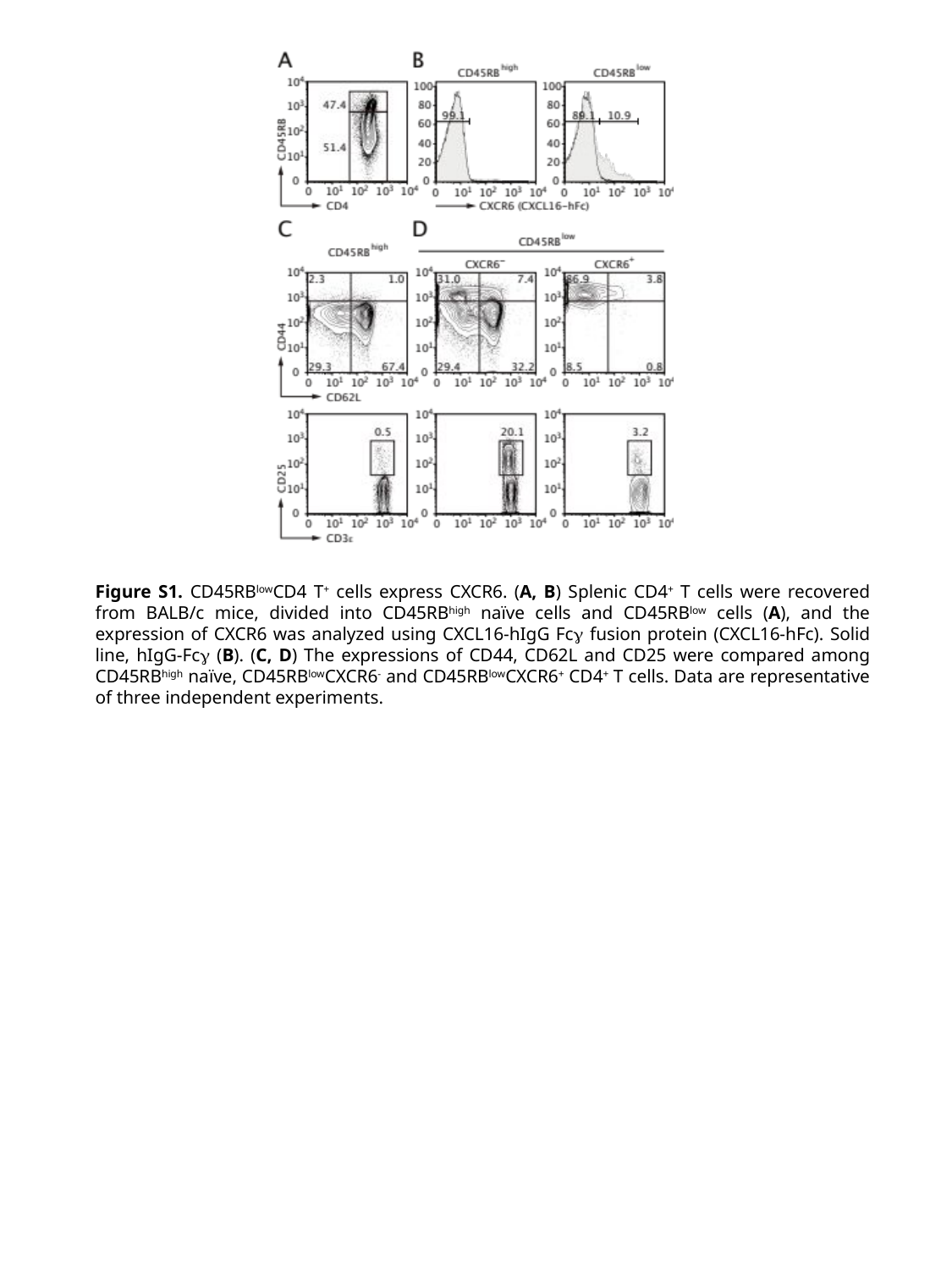

Figure S1. CD45RBlowCD4 T+ cells express CXCR6. (A, B) Splenic CD4+ T cells were recovered from BALB/c mice, divided into CD45RBhigh naïve cells and CD45RBlow cells (A), and the expression of CXCR6 was analyzed using CXCL16-hIgG Fcg fusion protein (CXCL16-hFc). Solid line, hIgG-Fcg (B). (C, D) The expressions of CD44, CD62L and CD25 were compared among CD45RBhigh naïve, CD45RBlowCXCR6- and CD45RBlowCXCR6+ CD4+ T cells. Data are representative of three independent experiments.
