# Supplementary material for: Distinct Roles for CXCR6+ and CXCR6− CD4+ T Cells in the Pathogenesis of Chronic Colitis
Source: PLoS One. 2013 Jun 19;8(6):e65488. doi: 10.1371/journal.pone.0065488 (PMC3686755; doi:10.1371/journal.pone.0065488)
Supplement: Figure S2 — SP and MLN CXCR6 − and CXCR6+CD4+ T-cell subsets were analyzed for expression of activation and memory markers on week 8 post-transfer of naïve CD4+ T cells. (A–C) The each subset was gated into CD127−CD62L−CD27−CD43+CD44+ to measure the proportion of effector T cells (a). (D–G) Memory population (CD44+CD127+) was subdivided using CD62L, CD27 and CD43 to measure late effector memory cells (CD62L−CD27−CD43+, b) early effector memory cells (CD62L−CD27+CD43+, C) and central memory cells (CD62L+CD27+, d). Data are representative of three independent experiments. (H) The relative percentages of effector, early effector memory and late effector memory cells in each subset are shown in a pie chart based on (A–G). (PPTX) [file pone.0065488.s002.pptx]

## Slide 1
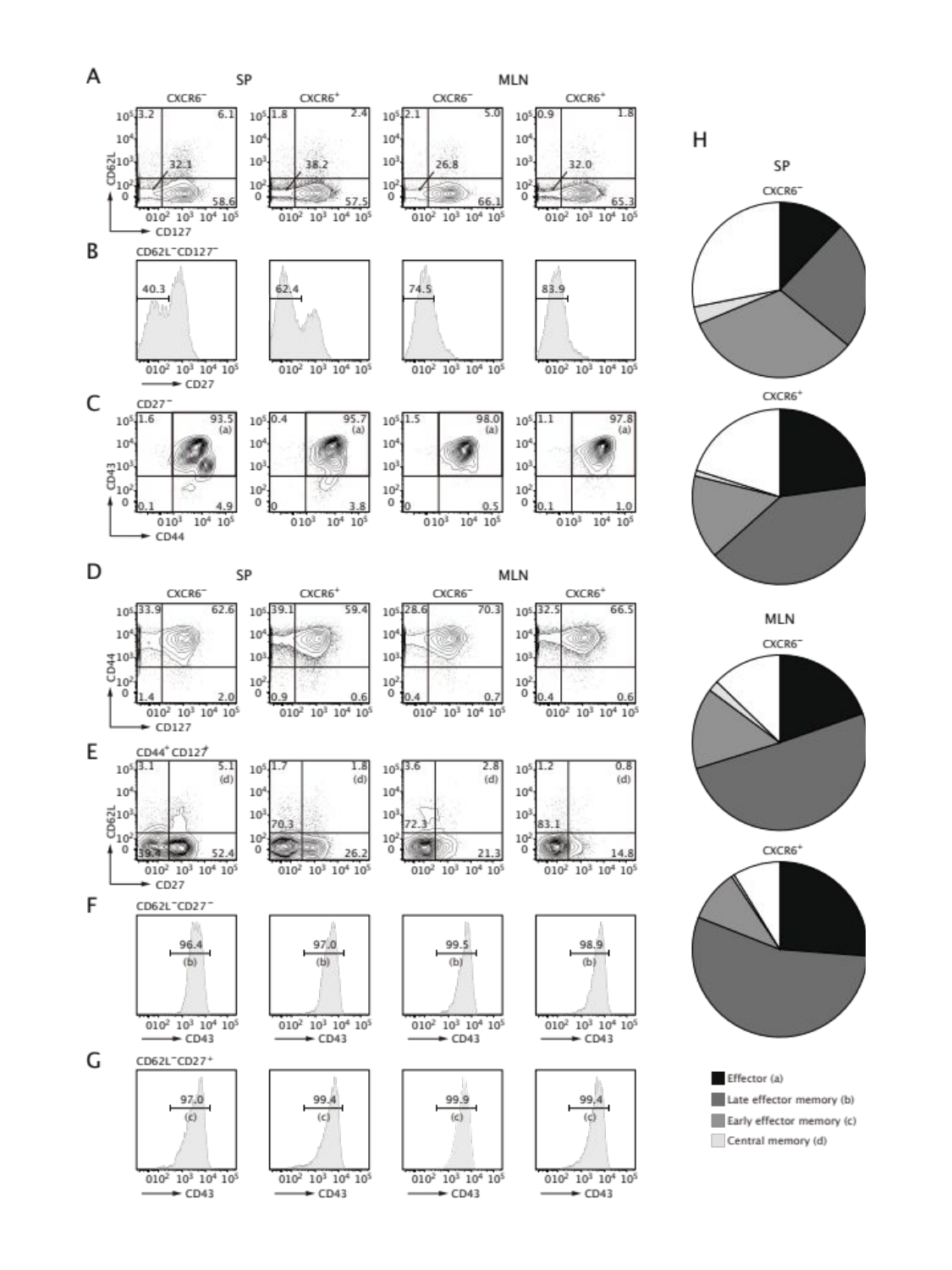

## Slide 2
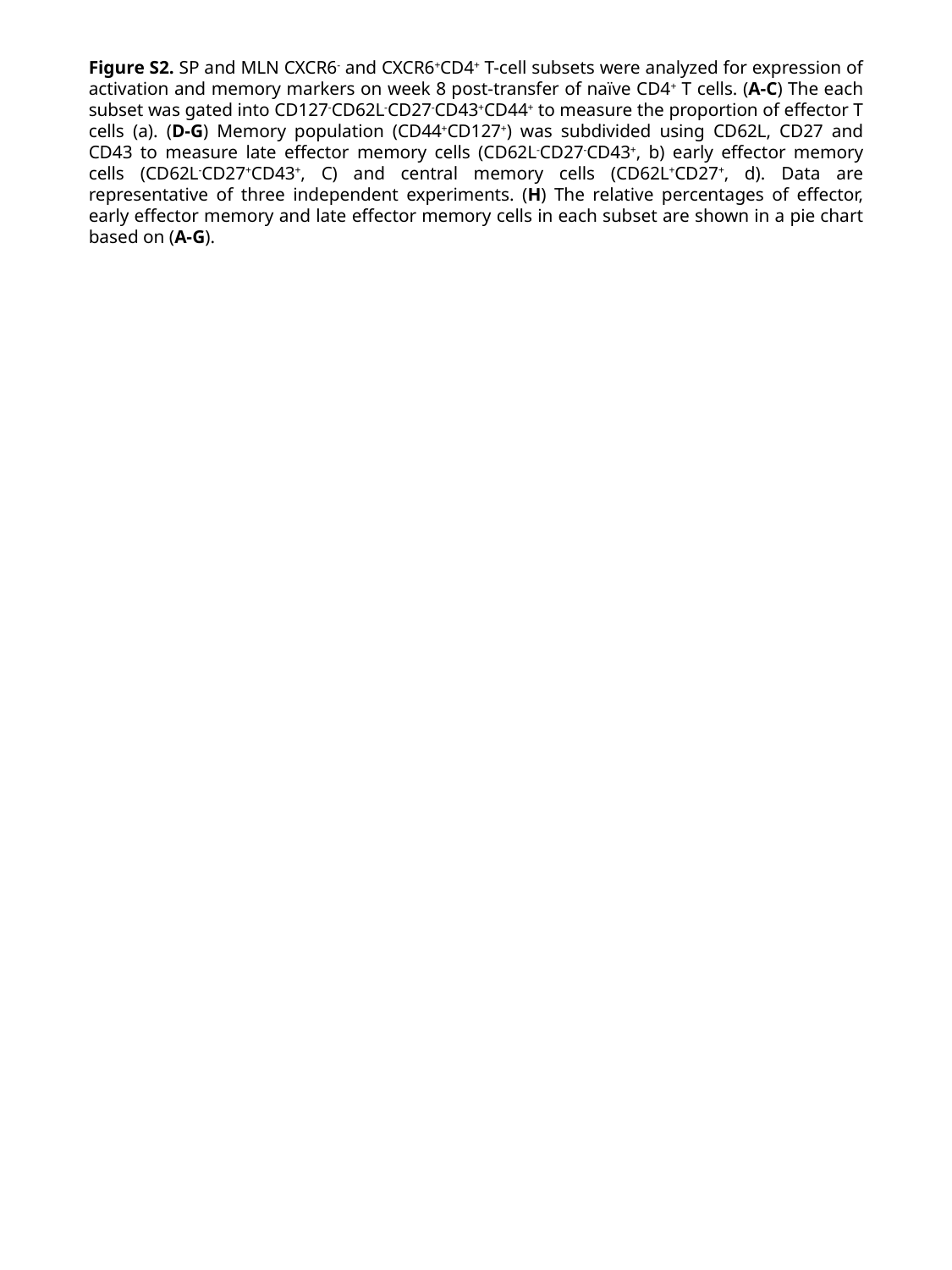

Figure S2. SP and MLN CXCR6- and CXCR6+CD4+ T-cell subsets were analyzed for expression of activation and memory markers on week 8 post-transfer of naïve CD4+ T cells. (A-C) The each subset was gated into CD127-CD62L-CD27-CD43+CD44+ to measure the proportion of effector T cells (a). (D-G) Memory population (CD44+CD127+) was subdivided using CD62L, CD27 and CD43 to measure late effector memory cells (CD62L-CD27-CD43+, b) early effector memory cells (CD62L-CD27+CD43+, C) and central memory cells (CD62L+CD27+, d). Data are representative of three independent experiments. (H) The relative percentages of effector, early effector memory and late effector memory cells in each subset are shown in a pie chart based on (A-G).
