# Supplementary material for: Distinct Roles for CXCR6+ and CXCR6− CD4+ T Cells in the Pathogenesis of Chronic Colitis
Source: PLoS One. 2013 Jun 19;8(6):e65488. doi: 10.1371/journal.pone.0065488 (PMC3686755; doi:10.1371/journal.pone.0065488)
Supplement: Figure S3 — Well-proliferated CD4+ T cells express CXCR6, and their CXCR6 expression is correlated with IFN-g and IL-2 productions. CFSE-labeled naïve CD4+ T cells were differentiated under Th1 condition. On the 6 days of culture, the CXCR6 expression and cytokine production were determined by flow cytometry. (PPTX) [file pone.0065488.s003.pptx]

## Slide 1
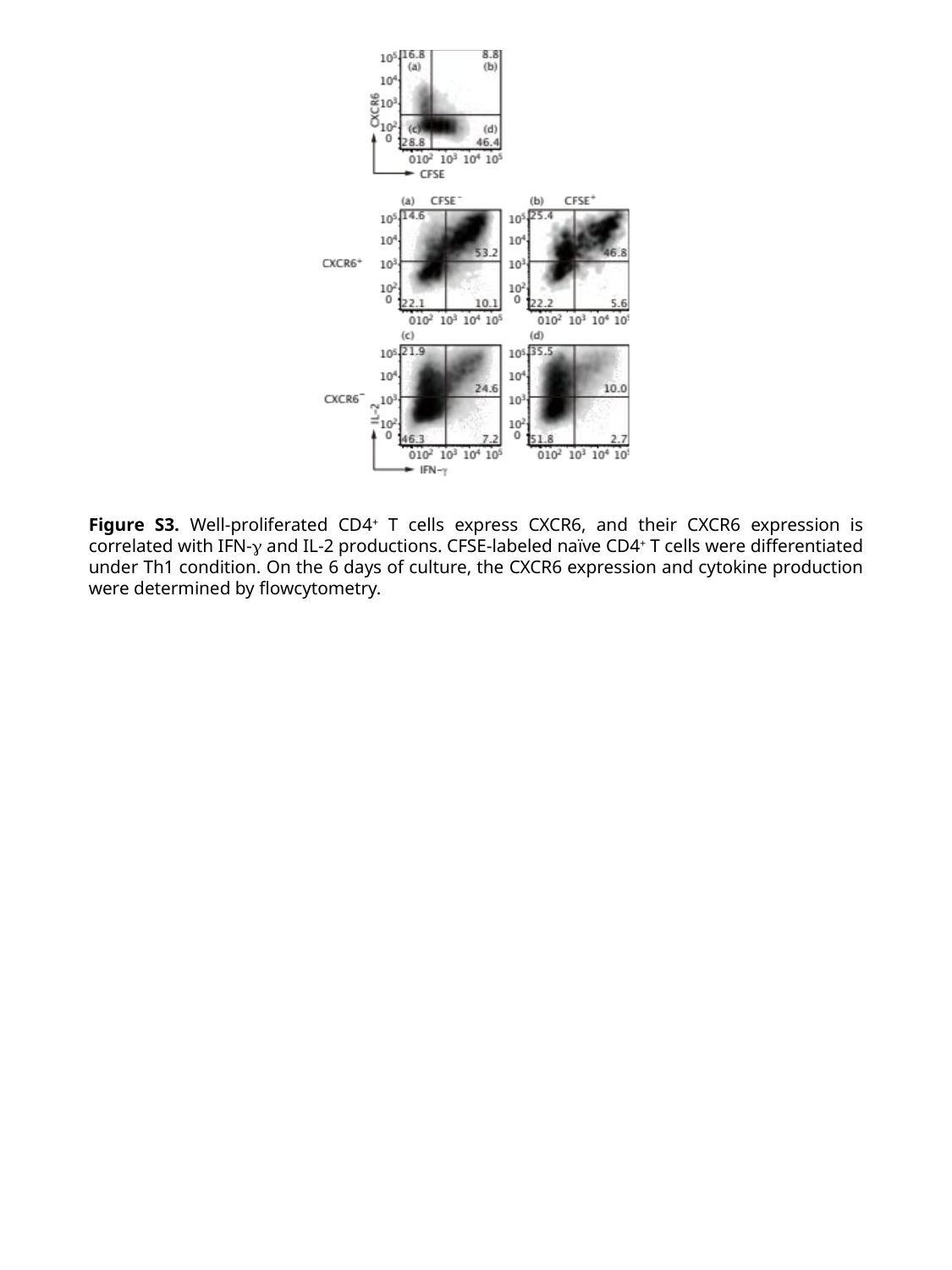

Figure S3. Well-proliferated CD4+ T cells express CXCR6, and their CXCR6 expression is correlated with IFN-g and IL-2 productions. CFSE-labeled naïve CD4+ T cells were differentiated under Th1 condition. On the 6 days of culture, the CXCR6 expression and cytokine production were determined by flowcytometry.
