# Supplementary material for: Distinct Roles for CXCR6+ and CXCR6− CD4+ T Cells in the Pathogenesis of Chronic Colitis
Source: PLoS One. 2013 Jun 19;8(6):e65488. doi: 10.1371/journal.pone.0065488 (PMC3686755; doi:10.1371/journal.pone.0065488)
Supplement: Figure S4 — CXCR6+ cells express IL-17A in CD colitis. Immunohistochemistry of CXCR6 (left) and IL-17A (right) was performed on serial paraffin sections of colonic mucosa from patients with CD. (PPTX) [file pone.0065488.s004.pptx]

## Slide 1
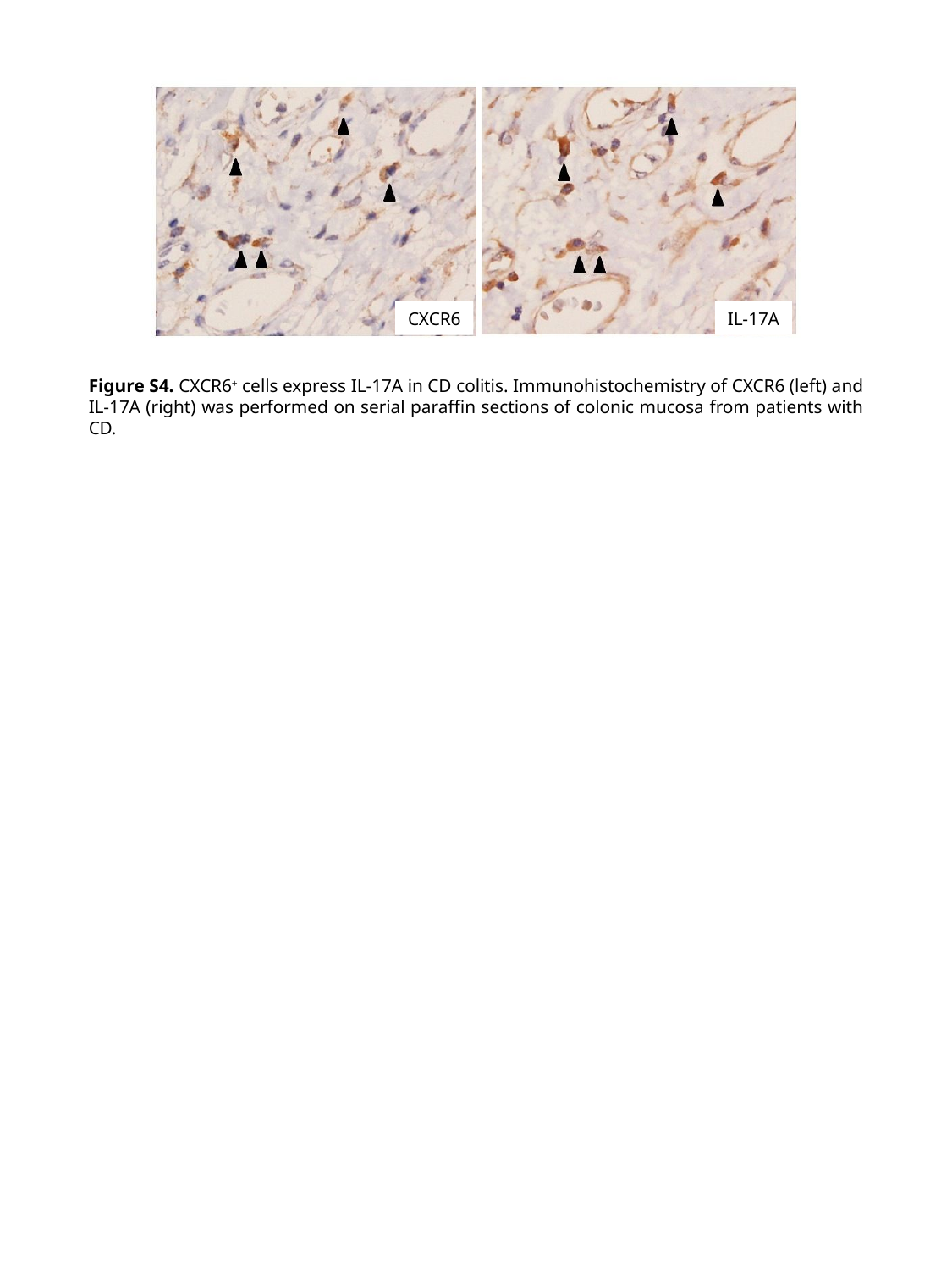

CXCR6
IL-17A
Figure S4. CXCR6+ cells express IL-17A in CD colitis. Immunohistochemistry of CXCR6 (left) and IL-17A (right) was performed on serial paraffin sections of colonic mucosa from patients with CD.
