# Supplementary material for: Distinct Roles for CXCR6+ and CXCR6− CD4+ T Cells in the Pathogenesis of Chronic Colitis
Source: PLoS One. 2013 Jun 19;8(6):e65488. doi: 10.1371/journal.pone.0065488 (PMC3686755; doi:10.1371/journal.pone.0065488)
Supplement: Figure S5 — CXCL16 stimulation does not enhance effector cytokine production. Naïve CD4+ T cells were differentiated under Th1 condition with or without soluble CXCL16 or plate-bound CXCL16-human IgG-Fc fusion protein (CXCL16-hFc). Human IgG-Fc (hFc) was used as a control for CXCL16-Fc. After 6 days of culture, cytokine production in CXCR6+ subset was examined by flow cytometry. (PPTX) [file pone.0065488.s005.pptx]

## Slide 1
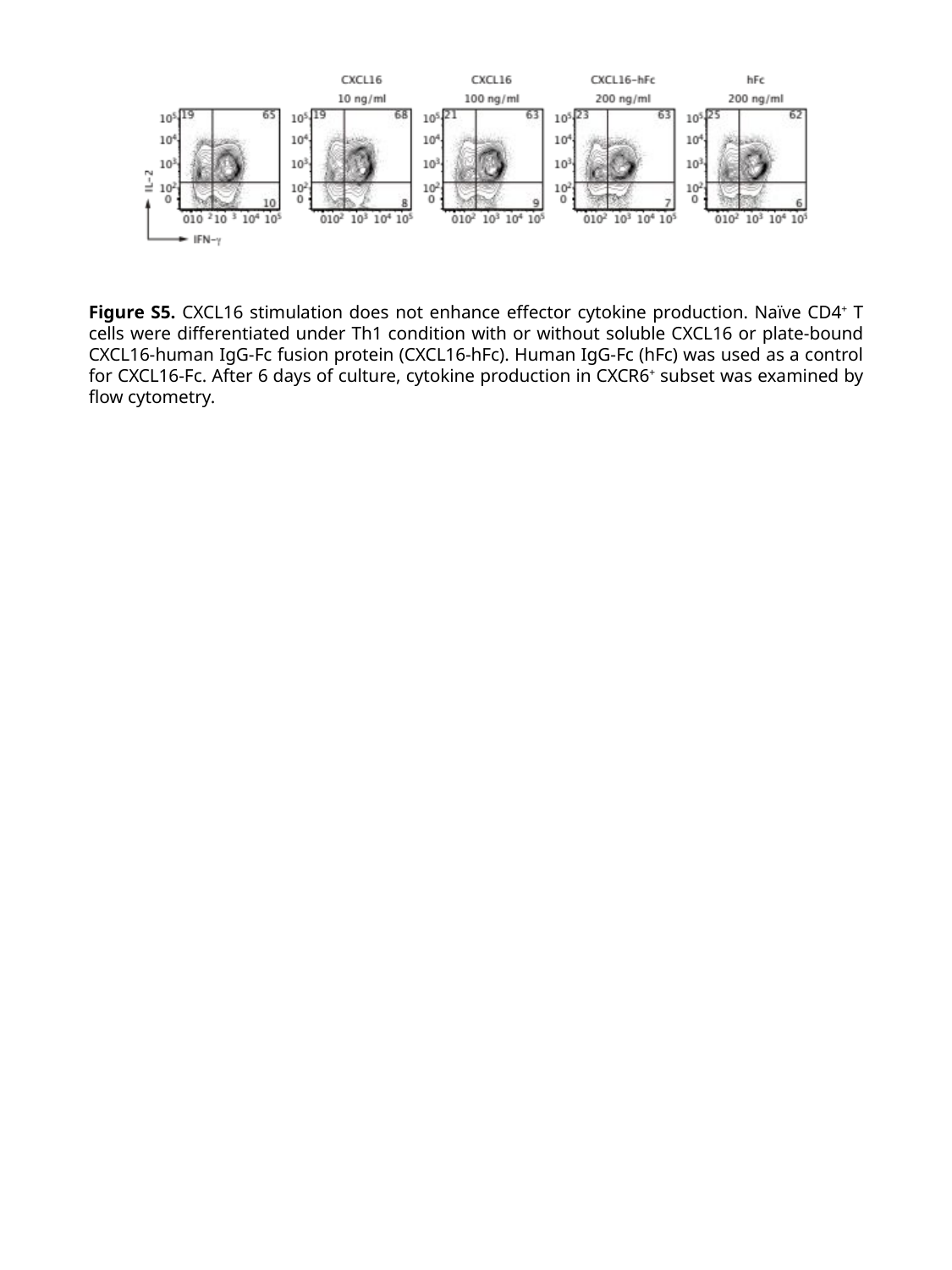

Figure S5. CXCL16 stimulation does not enhance effector cytokine production. Naïve CD4+ T cells were differentiated under Th1 condition with or without soluble CXCL16 or plate-bound CXCL16-human IgG-Fc fusion protein (CXCL16-hFc). Human IgG-Fc (hFc) was used as a control for CXCL16-Fc. After 6 days of culture, cytokine production in CXCR6+ subset was examined by flow cytometry.
