# Supplementary material for: Distinct Roles for CXCR6+ and CXCR6− CD4+ T Cells in the Pathogenesis of Chronic Colitis
Source: PLoS One. 2013 Jun 19;8(6):e65488. doi: 10.1371/journal.pone.0065488 (PMC3686755; doi:10.1371/journal.pone.0065488)
Supplement: Table S1 — (PPTX) [file pone.0065488.s006.pptx]

## Slide 1
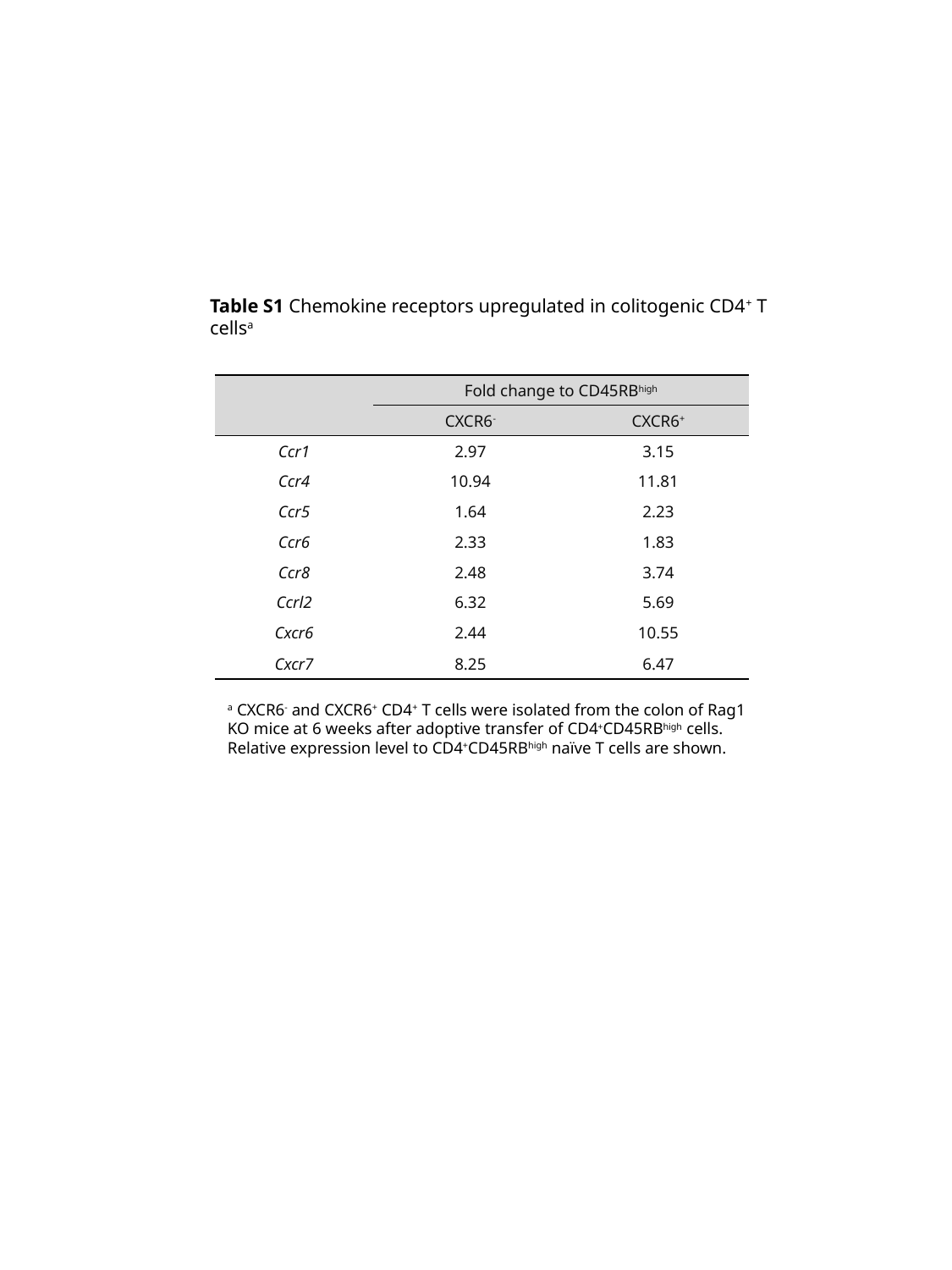

Table S1 Chemokine receptors upregulated in colitogenic CD4+ T cellsa
| | Fold change to CD45RBhigh | |
| --- | --- | --- |
| | CXCR6- | CXCR6+ |
| Ccr1 | 2.97 | 3.15 |
| Ccr4 | 10.94 | 11.81 |
| Ccr5 | 1.64 | 2.23 |
| Ccr6 | 2.33 | 1.83 |
| Ccr8 | 2.48 | 3.74 |
| Ccrl2 | 6.32 | 5.69 |
| Cxcr6 | 2.44 | 10.55 |
| Cxcr7 | 8.25 | 6.47 |
a CXCR6- and CXCR6+ CD4+ T cells were isolated from the colon of Rag1 KO mice at 6 weeks after adoptive transfer of CD4+CD45RBhigh cells. Relative expression level to CD4+CD45RBhigh naïve T cells are shown.
